# Automated Metrics for the Diagnosis of Instability Between the 2nd and 7th Cervical Vertebrae

**DOI:** 10.3390/bioengineering13030258

**Published:** 2026-02-24

**Authors:** John Hipp, Charles Reitman, Christopher Chaput, Mathew Gornet, Trevor Grieco

**Affiliations:** 1 Medical Metrics, Houston, TX 77057, USA; 2 Department of Orthopaedic Surgery and Rehabilitation, Medical University of South Carolina, Charleston, SC 29425, USA; reitman@musc.edu; 3 Department of Orthopaedic Surgery, University of Texas Health Science Center at San Antonio, San Antonio, TX 78229, USA; chaput@uthscsa.edu; 4 The Orthopedic Center of St. Louis, Chesterfield, MO 63017, USA

**Keywords:** cervical spine instability, subaxial, diagnosis, radiographic, whiplash-associated disorders, ligamentous injury, alteration of motion segment integrity (AOMSI)

## Abstract

Diagnosing cervical spine instability with flexion-extension radiographs is challenging, as current guidelines are based on limited cadaver studies and do not adequately account for level, vertebral size, or patient effort. There is a need for automated cervical instability metrics anchored to normative reference data, accompanied by evidence on how often abnormal findings occur in real clinical populations and which soft-tissue injury patterns they can detect. We developed and evaluated fully automated, radiographic-based cervical intervertebral motion (IVM) metrics—adapted from prior lumbar methods—using an FDA-cleared analysis pipeline that segments C2–C7 and derives rotation, translation, disc heights, and regression-based instability indices. Normative reference data were first established from flexion-extension radiographs of 341 asymptomatic volunteers after excluding radiographically degenerated levels. Abnormality prevalence was then estimated in two symptomatic cohorts: pooled preoperative clinical-trial radiographs and 881 patients with symptoms attributed to motor-vehicle accidents, excluding levels with <5° rotation to reduce unreliable data due to insufficiently stressed spines. Finally, potential diagnostic performance was assessed in a controlled cadaveric ligament-sectioning model (12 cadavers) using ROC analysis and Youden’s J thresholds. Across clinical cohorts, objective IVM abnormalities were uncommon. Prevalence increased when studies demonstrated adequate total C2–C7 motion, emphasizing the importance of patient effort. In cadavers, vertical instability metrics were most discriminative (AUC 0.96–0.97) with high sensitivity (0.89) and perfect specificity at optimal thresholds, whereas translation changed minimally with sectioning. These results support regression-based instability indices as promising candidates for standardized, physiology-guided cervical instability assessment.

## 1. Introduction

Prior publications have emphasized the clinical importance of accurately diagnosing cervical spine instability [1,2,3,4,5]. Commonly used radiographic criteria trace back to a 1975 study in which anterior-to-posterior and posterior-to-anterior sequential ligament sectioning was performed in 17 cadaveric motion segments stripped of all surrounding muscles [1,6]. By contemporary standards, the experimental methods were rudimentary, the sample size was small, and there was no demonstration that stepwise sectioning accurately reproduces the biomechanical consequences of real traumatic injuries. Moreover, such cadaveric preparations provide little insight into instability arising from degenerative processes, which are believed to be important contributors to clinical cervical pathology in some patients. As a result, despite widespread clinical interest, there remains no well-validated, reliable, practical, image-based diagnostic test for cervical spine instability [7,8]. Collectively, the available evidence highlights a substantial unmet need and supports the pursuit of more precise and reproducible imaging-based methods for identifying clinically meaningful cervical instability.

### Hypothesized Requirements for Cervical Spine Instability Diagnostic Tests

Building on prior evidence and established principles governing the accuracy of objective radiographic measurements [9,10], we propose that reliable diagnostic tests for cervical instability should meet the following requirements:

1. Use imaging that is widely accessible, low-cost, and of acceptably low radiation dose [11,12].
2. Employ standardized and easily reproducible imaging protocols that consistently stress the cervical spine sufficiently to reveal incompetent motion restraints [13,14,15,16].
3. Incorporate robust normative reference data capable of distinguishing normal from abnormal intervertebral motion [17].
4. Adjust for anatomical variability across individuals, including differences in vertebral size, and account for variable radiographic magnification [6,18,19,20].
5. Account for variability in the magnitude of flexion and extension so that measured abnormalities reflect pathology rather than patient effort or positioning [13,21,22].
6. Produce diagnostic metrics that can be rapidly generated and easily interpreted in clinical settings [23,24].
7. Demonstrate sensitivity to disc and ligament injuries known to produce mechanical instability [25,26,27,28].
8. Demonstrate clinical utility, meaning that the metrics meaningfully improve diagnostic accuracy and help guide treatment decisions [29,30].

The purpose of this research is to develop and evaluate new fully automated cervical spine instability metrics—adapted from previously published lumbar methods—that satisfy some of these requirements. In addition to exploring new metrics, traditional metrics were also assessed, including those detailed in the American Medical Association Guides to the Evaluation of Impairment [31]. Using flexion-extension radiographs from asymptomatic volunteers, symptomatic patient populations, and a cadaveric ligament-sectioning model, the goal was to establish normative reference data, estimate the prevalence of abnormalities in clinical cohorts, and assess the potential diagnostic performance of metrics for detecting injury-induced instability between C2 and C7. To our knowledge, no prior study has integrated normative data, symptomatic patient prevalence, and cadaveric validation into a single methodological framework.

## 2. Materials and Methods

Cervical spine flexion-extension X-rays were used to:

(1) Establish reference data to differentiate between normal and abnormal IVM
(2) Assess the prevalence of IVM abnormalities in patient populations
(3) Assess the effects of sequential ligament sectioning on IVM

**Figure 1:**
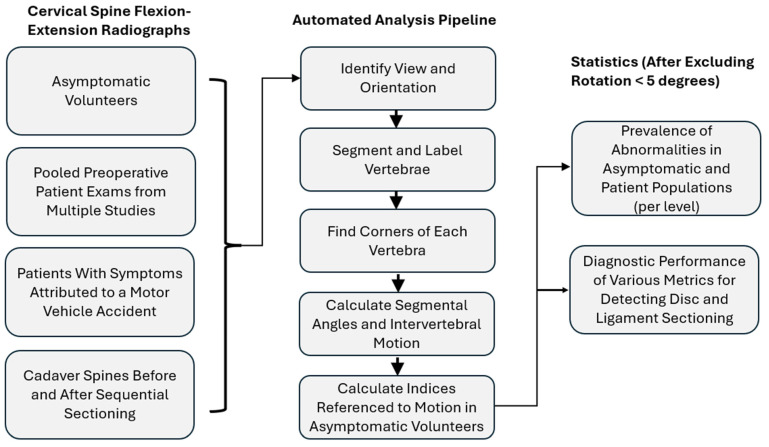
Flow diagram to summarize the methods.

Figure 1 provides a flow diagram for the methods.

### 2.1. Flexion-Extension Exams for IVM Reference Data

We established intervertebral motion (IVM) reference data through a retrospective analysis of flexion-extension radiographs from a previously reported study of asymptomatic volunteers [19]. The research protocol (BCM IRB H-10288) enrolled volunteers who provided informed consent, reported no history of cervical spine disorders or significant neck pain, and performed maximum voluntary flexion and extension during imaging. Degeneration from C2–C3 through C6–C7 was graded independently by two experienced radiologists using the Kellgren–Lawrence system [32]; discrepancies were adjudicated by a third reader. Normative reference data were derived after excluding levels with definite radiographic degeneration. In addition, levels with intervertebral rotation < 5° were excluded. This <5° exclusion was derived from a qualitative synthesis of published neutral-zone data [33,34,35,36]. It was selected to remove flexion-extension examinations that did not impose sufficient motion to activate the intrinsic stabilizing structures (including the disc, ligaments, and facet joints). The goal was to restrict analysis to studies in which normally functioning restraints would be expected to limit motion within physiologic bounds.

Standardized disc height (SDH) was calculated for each level as the number of standard deviations by which the level’s average disc height differed from the asymptomatic reference mean. Average disc height (AvgDH) was defined as the mean of the anterior and posterior disc heights measured in both flexion and extension. SDH was computed as:

SDH = (AvgDH_subject − AvgDH_reference_mean)/SD_reference.

### 2.2. Flexion-Extension Exams to Assess the Prevalence of IVM Abnormalities in Patient Populations

Two patient populations were studied: (1) Pooled preoperative clinical trial radiographs representative of a diverse population of symptomatic patients, though without clinical detail. (2) A whiplash cohort representing a more homogeneous group with a somewhat more consistent injury mechanism.

#### 2.2.1. Pooled Image Analysis

Preoperative lateral flexion-extension radiographs from multiple clinical studies were retrospectively analyzed to estimate the prevalence of intervertebral motion and alignment abnormalities in symptomatic patients. All images were obtained from a quality-control database maintained by an independent imaging core laboratory (Medical Metrics, Inc., Houston, TX, USA). The radiographs were fully anonymized prior to analysis, and no associated diagnostic or treatment information was available; only that patients were symptomatic and imaged before any intervention. Accordingly, these data permit only broad estimates of abnormality prevalence. The retrospective analysis of anonymized images using fully automated methods was determined by Pearl IRB to be exempt under 45 CFR 46.104(d)(4) (“Secondary Research Uses of Data or Specimens”). The prevalence of IVM abnormalities was determined after excluding levels with <5° of rotation.

#### 2.2.2. Analysis of Patients with Whiplash-Associated Disorders

Flexion-extension radiographic examinations were obtained for 881 patients who sought care from a single spine surgeon for symptoms attributed, at least in part, to a motor vehicle accident (MVA). Imaging was performed a mean of 245 ± 420 days after the accident (range, 1–5608 days). All patients provided informed consent for the use of anonymized imaging data for research. Exams were acquired during routine clinical practice between July 2011 and February 2019 and were retrospectively analyzed. Inclusion criteria were (1) documentation of an MVA in the medical history that the patient believed contributed to their symptoms, and (2) availability of cervical spine flexion-extension radiographs. These examinations represent a real-world sample of typical clinical presentations. The prevalence of IVM abnormalities was determined. Prevalence estimates excluded levels with <5° of intervertebral rotation.

### 2.3. Flexion-Extension Exams to Assess the Effect of Ligament Sectioning on IVM

Flexion-extension radiographic examinations from a previously reported cadaveric study were reanalyzed using automated methods. This provides a controlled injury model against which candidate diagnostic metrics can be compared. In the original study, flexion-extension images were acquired before and after sequential ligament sectioning performed from anterior to posterior [37]. Twelve post–rigor mortis (and not embalmed) whole human cadavers (seven female, five male; age 80 ± 9.3 years) were positioned in a seated posture and secured to a chair. Controlled flexion and extension of the head and neck were produced using a helmet-mounted loading apparatus. The present analysis evaluated the sensitivity and specificity of intervertebral motion (IVM) metrics for detecting abnormalities induced at each sectioning stage and identified optimal diagnostic thresholds using Youden’s J Statistic to analyze receiver operating characteristic (ROC) curves, implemented in Python (version 3.11) using the scikit-learn (version 1.8.0) metrics package. For each candidate cutoff value, we calculated sensitivity and specificity and then computed Youden’s J as: J = sensitivity + specificity−1. The cutoff that maximized J was selected as the “optimal” diagnostic threshold because it maximizes the vertical distance between the ROC curve and the chance line, thereby maximizing overall correct classification while weighting sensitivity and specificity equally.

### 2.4. Automated Analysis of Flexion-Extension X-Rays

Flexion-extension radiographs from all studies were analyzed using a single FDA-cleared system (SpineCAMP^®^, Medical Metrics, Inc., Houston, TX, USA). The system employs a fully automated pipeline incorporating neural networks and rule-based logic to segment and label vertebrae from C2 to C7 and to identify standardized vertebral corner landmarks representing the midsagittal plane of each vertebra [38]. The resulting fully automated landmarks and intervertebral motion (IVM) metrics are equivalent to those produced by the semi-automated Quantitative Motion Analysis (QMA) method used in multiple prior investigations [39,40].

### 2.5. Calculation of Basic Metrics from Anatomic Landmarks

**Figure 2:**
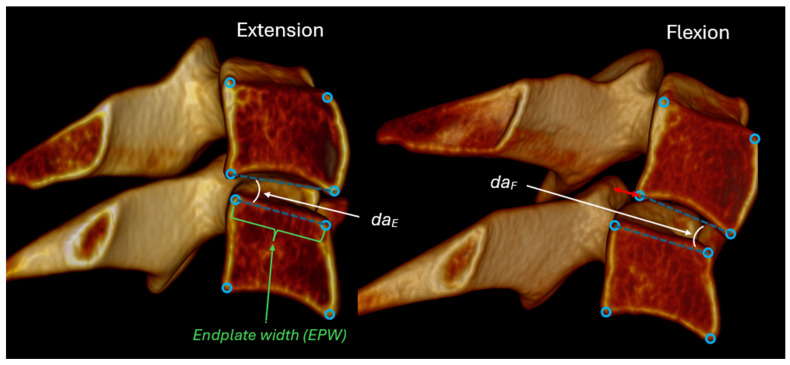
Details of the methods used to calculate disc angles in flexion and extension. Intervertebral rotation is calculated from the difference in disc angles. Intervertebral translation (red arrow) is measured at the posterior-inferior corner of the superior vertebra in a direction defined by the superior endplate of the inferior vertebra. Intervertebral translation is normalized to the superior endplate width of the inferior vertebra.

**Figure 3:**
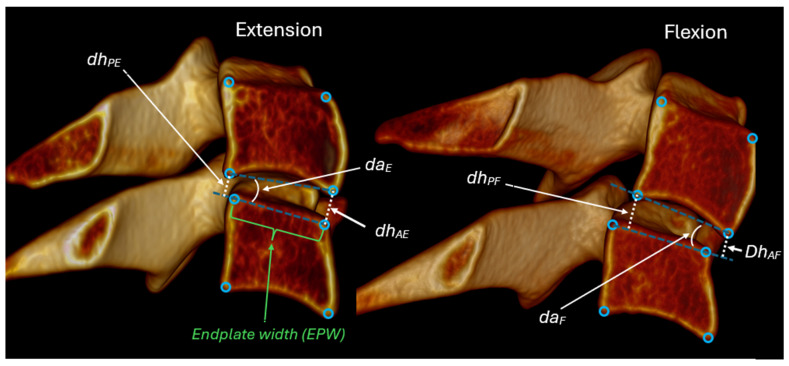
Anterior and posterior disc heights (dh_A_ and dh_P_) are measured in flexion and extension as indicated in this figure. The change in disc height between flexion and extension is used to calculate the anterior- and posterior-vertical instability indices.

All intervertebral metrics were derived from four standardized anatomic landmarks identified on each vertebra from C2 to C7 in both flexion and extension radiographs. Sagittal plane intervertebral rotation and translation were measured (Figure 2), as were anterior and posterior disc heights, and disc angles (Figure 3). The difference between the segmental angle at a specific level relative to the segmental angles at adjacent levels was also calculated to enable testing for an 11-degree difference between levels as described in the AMA impairment rating guide [31]. Translations and disc heights were expressed as a percentage of endplate width to avoid magnification-related errors and to account for inter-individual variability in vertebral size [6,18,19,20].

Translation was also reported in millimeters by assigning each level an average endplate width, determined from a prior analysis of several hundred radiographs that included a calibrated scaling device [10]. Segment angles were additionally calculated using the inferior endplate of the superior vertebra and the inferior endplate of the adjacent inferior vertebra, consistent with measurement conventions illustrated in the AMA Guides to the Evaluation of Permanent Impairment [7]. Rotation and translation guidelines described in the AMA Guides to the Evaluation of Permanent Impairment were analyzed. These guides are widely adopted or mandated by most U.S. state workers’ compensation systems, federal schedules (e.g., FECA, Longshore), and many courts for determining permanent disability awards and settlement values (https://aaeme.com/wp-content/uploads/2024/04/Use-of-the-AMA-Guides-by-State-1.pdf (accessed on 19 November 2025).

### 2.6. Normative Reference Data for the Asymptomatic Population

Means and standard deviations for intervertebral rotation and translation were calculated for all radiographically normal levels. To facilitate comparison with criteria used in the AMA Guides for assessing alteration of motion segment integrity (AOMSI), the difference in rotation at each level relative to its immediately adjacent levels was computed. Because some sources assess differences in static segment angles across levels, the difference in segment angles between each level and its adjacent levels was also calculated. When both C2 and C7 were visible on flexion and extension radiographs, the total C2–C7 sagittal plane rotation was measured, and intervertebral rotations were expressed as a percentage of this global rotation.

Following methods established for the lumbar spine [41], linear regressions were used to obtain the slope (M_RDT_) and Y-Intercept (B_RDT_) needed to calculate Rotation Dependent Translation (RDT) for each level. In addition, the slope (M_RDDW_) and Y-Intercept (B_RDDW_) needed to calculate Rotation Dependent Disc Widening (RDDW) were determined for each level. The standard error of the forecast (SEF) was calculated for RDT and RDDW. The SEF was used instead of the standard deviation because it provides a point-specific estimate of variability at a given rotation, rather than a single estimate for all rotations. The SEF was calculated using Stata version 15 by approximating the upper and lower bounds of the data variability as a quadratic. Regression coefficients and SEF values were then used to compute the Translational Instability (TI) Index and the Anterior and Posterior Vertical Instability (AVI, PVI) Indices.

$$TI\;Index=\frac{Translation-\left(Rotation\;*\;M_{RDT}+B_{RDT}\right)}{{SEF}_{RDT}}$$

$$AVI\;Index=\frac{({ADH}_{Ext}-{ADH}_{Flex})-\left(Rotation\;*\;M_{RDDW}+B_{RDDW}\right)}{{SEF}_{RDDW}}$$

$$PVI\;Index=\frac{({PDH}_{Flex}-{PDH}_{Ext})-\left(Rotation\;*\;M_{RDDW}+B_{RDDW}\right)}{{SEF}_{RDDW}}$$

ADH = Anterior Disc Height; PDH = Posterior Disc Height; Ext = Extension; Flx = Flexion.

RDT = Rotation Dependent Translation; RDDW = Rotation Dependent Disc Widening.

Values between −2 and +2 were considered within normal limits. Use of these standardized indices eliminates the need for level-dependent reference tables, enables pooling of data across levels, and simplifies clinical interpretation. Vertical instability quantifies the amount of change in disc height that occurs with rotation. Abnormally great change in the intervertebral disc height (for the amount of rotation) can indicate disc laxity or an inability of the ligaments and/or disc to maintain disc opening and closing to within normal limits, and this may indicate disruption of intervertebral motion restraints [41,42,43,44].

## 3. Results

Not all vertebrae were analyzable in every radiograph due to limited field of view, shoulder-related scatter, radiographic noise, or suboptimal projection/exposure.

### 3.1. IVM Reference Data

Table 1 summarizes the means and standard deviations for radiographically normal levels in asymptomatic volunteers. All metrics differed significantly across levels (*p* < 0.0001) and were used to inform abnormality assessments in the patient cohorts. As expected, not all levels (C2–C7) were visible in all radiographs; C7 was most frequently obscured due to shoulder-related radiographic noise. Among radiographs in which both C2 and C7 were analyzable in flexion and extension, the total C2–C7 rotation was 73.9° ± 14.0°. Segmental angle differences were calculated from the flexion images as the maximum difference between each level and its adjacent levels. Table 1 also provides the differences between levels in intervertebral rotation and segment angles (measured from the flexion X-rays). These data are intended to support a critique of the radiographic criteria described in the various editions of the AMA guidelines for assessing alteration of motion segment integrity (AOMSI) [31,45,46].

The relationships between intervertebral rotation and translation, and between intervertebral rotation and changes in disc height, could be described using linear regressions at each level. Table 2 provides the R-squared statistics for the linear regressions relating rotation to translations and rotation to changes in disc heights. This is essential to the development of the TI-, AVI-, and PVI-indices.

**Figure 4:**
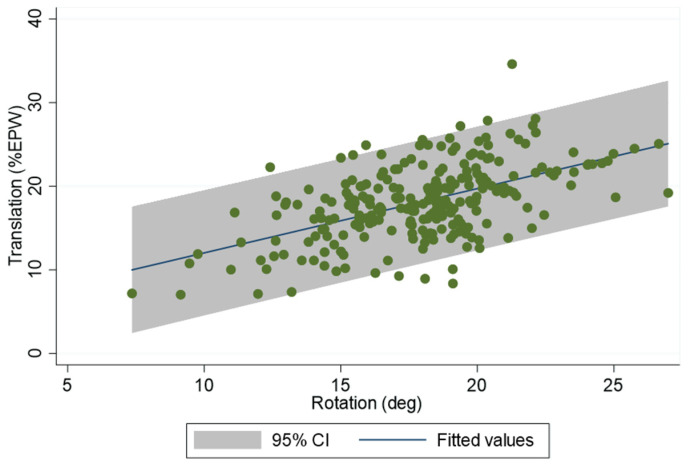
Relationship between translation, in units of % endplate width (EPW), and rotation at the C4–C5 level.

For the relationship between rotation and translation, the slopes were significantly different across levels (*p* < 0.0001), so linear regressions were fit for each level. Figure 4 shows the relationship between translation and rotation at the C4–C5 level. In contrast, the slopes of linear regressions between rotation and the change in anterior disc heights were very similar at all levels, so a single linear relation was established between rotation and the change in anterior disc heights. The same was true for the change in posterior disc heights.

**Figure 5:**
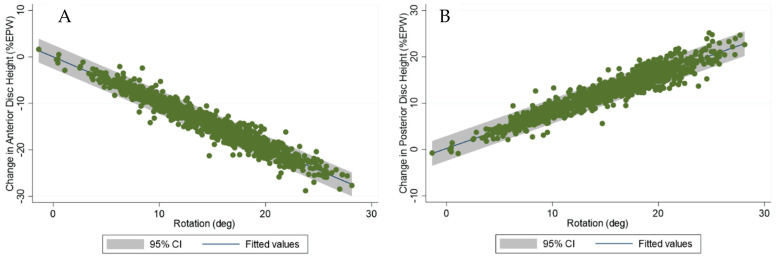
(**A**,**B**) Relationship between rotation (from extension to flexion) and the change in anterior and posterior disc heights, in units of percent endplate width (EPW), with all levels combined.

Figure 5A,B shows the relationship between the rotation (from extension to flexion) and the change in disc heights, with all levels combined.

### 3.2. Prevalence of IVM Abnormalities in Asymptomatic Volunteers

Table 3 reports the prevalence of abnormalities identified in the asymptomatic volunteers, using all spinal levels rather than limiting analyses to the radiographically normal levels used to derive normative reference data. These results demonstrate that detectable abnormalities can occur in asymptomatic individuals and provide context for interpreting prevalence estimates in patient populations.

Sagittal plane intervertebral rotation between flexion and extension was classified as abnormal when the difference between a level and its immediately adjacent levels exceeded 11° [1,31]. A difference in segmental angle greater than 11° on the flexion radiograph was also considered abnormal [7]. Intervertebral translation was classified as abnormal when it exceeded 20% of the vertebral anterior–posterior width [7].

The commonly cited criterion of >3.5 mm translation was not applied because it assumes radiographs with approximately 30% magnification. In contrast, our translation measurements were obtained in millimeters without magnification distortion, making this threshold inappropriate for our dataset.

### 3.3. Prevalence of IVM Abnormalities in Patient Populations

A total of 2134 preoperative cervical flexion-extension studies were available for analysis from multiple clinical research studies, though not every level could be analyzed in every exam. Table 4 presents the prevalence of abnormalities at the analyzable levels in the pooled exams. It is noteworthy that some prevalences are lower than in asymptomatic volunteers. In preoperative flexion-extension exams for symptomatic patients, the C2–C7 rotation was 45.9 ± 16.1 deg, compared with 73.9 ± 14.0 deg in asymptomatic volunteers, which could explain some of the differences in prevalence. Table 5 shows the prevalence when only flexion-extension exams with >60° of rotation between C2 and C7 are included. That threshold is approximately one standard deviation below the average C2–C7 rotation in asymptomatic volunteers. Note that in Table 5, the sample size is approximately 20% that in Table 4. This is due to some cases not showing both C2 and C7 in both flexion and extension images, and to rotation from C2 to C7 being ≤60°. The prevalences in Table 5 are more directly comparable to those in Table 3 because the C2–C7 rotation is better matched.

Table 6 presents the prevalence of abnormalities among patients seeking care from a spine surgeon for symptoms believed to be at least partially related to an MVA. The rotation from C2 to C7 was 60.5° ± 18.4° (for 460 patients for whom C2 to C7 could be analyzed).

### 3.4. Effects of Sequential Ligament Sectioning on IVM

**Figure 6:**
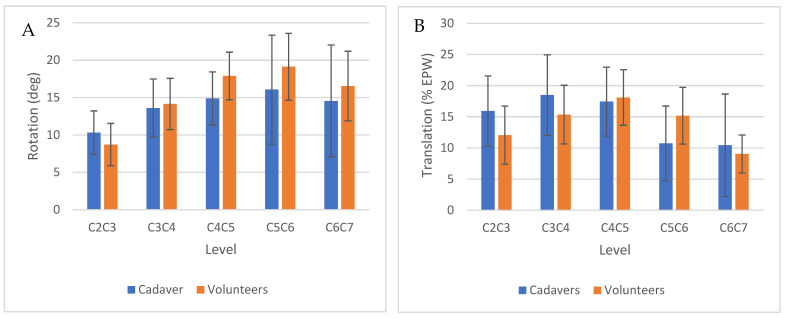
(**A**,**B**) Comparison of the intervertebral rotation at each level in the intact cadaver spine and in the asymptomatic volunteers.

**Figure 7:**
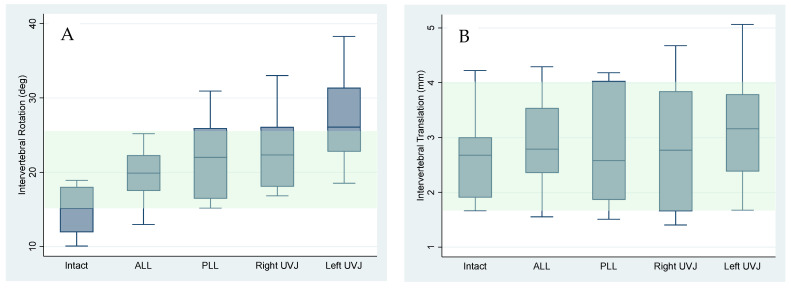
(**A**,**B**) Box and whisker plots illustrating intervertebral rotation and translation in the intact cadaver spines and after sequential sectioning of ligaments, disc, and uncovertebral joints (UVJ). The green-shaded regions show the normal range of rotation and translation at the C4–C5 level in the spines of asymptomatic volunteers.

**Figure 8:**
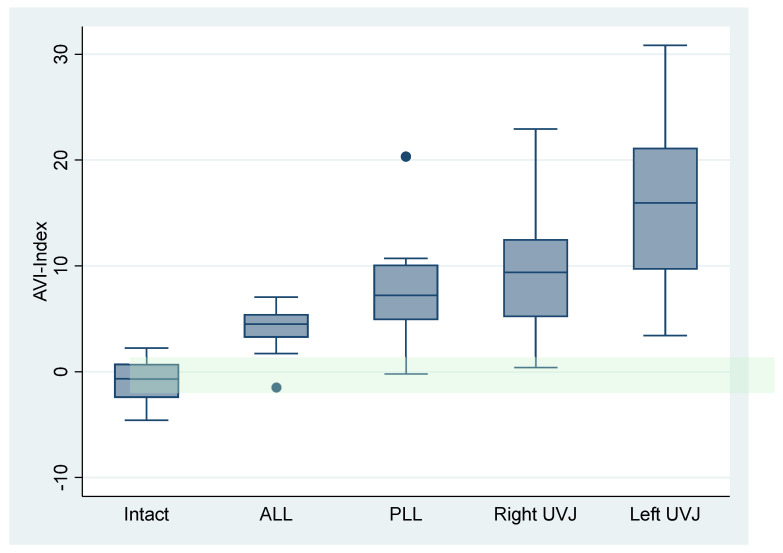
Box and whiskers plot illustrating the AVI-Index in the intact cadaver spines and after sequential sectioning of ligaments, disc, and uncovertebral joints (UVJ). The green-shaded region shows the normal range (−2 to +2) for spines in asymptomatic volunteers.

The intervertebral rotation and translation in the intact cadavers and asymptomatic volunteers were similar (*p* = 0.39 rotation, 0.17 translation, Figure 6A,B). Figure 7A,B show that, with sequential sectioning, intervertebral rotation increased (*p* = 0.0004), whereas intervertebral translation did not change significantly (*p* = 0.87). The TI-Index also did not change appreciably (*p* = 0.13). The difference in segment angle between C4–C5 and the adjacent levels, measured in the flexion X-rays, did not change significantly with sectioning (*p* = 0.21). The AVI index changed significantly with sectioning (Figure 8, *p* < 0.0001). The AVI and PVI indices were the only metrics that were sensitive and specific, even at the initial step of sequential sectioning. Based on Youden’s J statistic, the optimal threshold for AVI-Index to detect sectioning was 3.2, and at that threshold, the AVI-Index had a sensitivity of 0.89 and specificity of 1. Table 7 has the area under the curve (AUC), optimal thresholds, and sensitivity and specificity at the threshold for several potential instability metrics.

## 4. Discussion

Advancing spine care requires diagnostic tools that are not only accurate but also practical, reproducible, and can inform clinical interpretation when combined with clinical findings, across a broad spectrum of providers. The present study contributes several foundational steps toward this goal. The integration of normative reference data from asymptomatic volunteers, prevalence estimates from large and diverse patient cohorts, and validation against a controlled cadaveric injury model is intended to support prospective clinical research. This combined evidence base also highlights both the limitations of long-standing diagnostic criteria and the potential advantages of regression-based instability indices that adjust for level, patient effort, and vertebral size.

Importantly, these findings demonstrate a path toward harmonizing diagnostic reporting across surgical and non-surgical disciplines, reducing ambiguity in interpreting flexion-extension studies, and may help identify candidates for additional clinical assessment in future outcome-linked studies. Because flexion-extension radiographs are widely available, low-cost, and low-radiation, the metrics evaluated here could support scalable, standardized reporting workflows in future clinical implementations, contingent on prospective validation.

### 4.1. Prevalence of Abnormalities

A central finding of this work is the low prevalence of objective quantitative IVM abnormalities in symptomatic patients, where abnormal is defined as a metric outside of the 95% confidence intervals (Table 3, Table 4, Table 5 and Table 6). Since 95% confidence intervals were used as the reference for the TI, AVI, and PVI indices, “abnormal” motion will be found in a small proportion of asymptomatic volunteers (Table 3). Even among individuals evaluated before spine surgery (Table 4 and Table 5), or those presenting after motor-vehicle-related trauma (Table 6), most cervical motion segments exhibited values within normative limits when assessed using strictly defined rotation, translation, or vertical instability criteria. This is directionally consistent with the biological mandate of the cervical spine: to maintain stable alignment and protect the spinal cord and major neurovascular structures [2,3,47]. Because compromise of the stabilizing mechanisms in the cervical spine can produce severe neurologic or vascular injury, gross instability should be expected to be uncommon (2–8%) in living populations [1,48,49,50,51]. Careful use of validated instability metrics will likely prove that symptomatic instability occurs in some patients, and that quantitative measures cannot be used to diagnose cervical spine disorders exclusively. Cervical spine injuries are more common (42–50%) following severe or fatal blunt trauma [51,52,53].

The data also demonstrate that insufficient flexion-extension effort can obscure abnormalities. When analyses were restricted to studies with >60° of total C2–C7 rotation (Table 5)—approximately one standard deviation below the mean in asymptomatic volunteers—the prevalence of abnormalities increased meaningfully, particularly for PVI-Index and AMA AOMSI criteria. These findings reinforce that diagnostic tests relying on IVM must ensure the spine is adequately stressed, analogous to cardiac or pulmonary stress testing. Without sufficient motion, false-negative results are likely. An additional consideration is the possibility of muscle spasms restricting motion at some levels, or pain-guarding restricting motion at most levels. Both spasms and guarding could be important clinical findings, yet there are currently no validated and reliable diagnostic tests [54,55].

At the same time, some abnormalities are present in asymptomatic volunteers (Table 3). This parallels the interpretation of cervical degeneration on MRI: structural abnormalities become clinically meaningful only when concordant with the patient’s symptoms and biomechanical context [56,57]. Thus, future diagnostic frameworks should avoid treating any isolated abnormal metric as inherently pathological and instead integrate findings with clinical presentation and level-specific normative ranges.

### 4.2. Implications for Existing Instability Criteria

The present data provide an opportunity to critically examine the performance of widely used criteria for alteration of motion segment integrity (AOMSI). The AMA threshold of >11° segment angle difference between adjacent levels, derived from a small cadaveric study in 1975 [1], does not align consistently with the distribution of values in healthy volunteers (Table 1). For example, at C5–C6 and C6–C7, a 10° difference already falls outside the 95% confidence interval, suggesting that a single global threshold may misclassify levels.

Similarly, the AMA guideline that translation >20% of the vertebral width indicates instability is poorly aligned with live normative data. Nearly 28% of C4–C5 levels in asymptomatic volunteers exceeded this value (Table 3), demonstrating that anatomical and level-specific variability significantly influence translation magnitude. Use of the 20% translation guideline may lead to false-positive instability diagnoses, particularly when patients are encouraged to flex and extend maximally. The frequently cited threshold of 3.5 mm translation [58,59,60,61], which is based on an assumed 30% radiographic magnification, also proves problematic when magnification-free measurements are used. In the present dataset, 2.7 mm (the actual measured maximum translation found in the original cadaver study [1]) falls well within normal limits for many volunteers (Table 1). These inconsistencies highlight that legacy thresholds derived from limited cadaveric evidence are too coarse for clinical use in living populations.

Collectively, these observations indicate that future diagnostic systems must (1) incorporate level-specific reference values, (2) adjust for patient effort, and (3) account for vertebral size. Regression-based indices such as TI, AVI, and PVI meet these requirements and therefore represent promising candidates for next-generation instability assessment.

### 4.3. Diagnostic Performance of Candidate Metrics

The cadaveric ligament-sectioning model enables preliminary evaluation of the diagnostic accuracy of multiple metrics under controlled injury conditions. The AVI- and PVI-Indices demonstrated the highest discriminative performance, with AUC values of 0.96 and 0.97, respectively, and perfect specificity at the optimal Youden thresholds (Table 7). These indices were uniquely sensitive to early-stage destabilization—even the first step in the sectioning sequence—whereas rotation, translation, and segment angle differences showed limited sensitivity or poor AUC.

These findings suggest that vertical instability metrics may be the most biomechanically sensitive indicators of early ligamentous compromise, consistent with the understanding that ligamentous injury can alter intervertebral spacing before producing large rotational or translational displacements.

However, translation remained surprisingly unchanged through multiple sectioning stages (Figure 7B), indicating that sagittal-plane translation is not a reliable marker of acute ligamentous disruption under physiological loading. This finding is at odds with the common use of translation as a primary indicator of instability, particularly given that the IVM in the intact spines of cadavers was very similar to the IVM in live asymptomatic volunteers (Figure 6A,B). The fact that translation was not affected by anatomical sectioning might be due to the untouched facet joints, which maintained translational stability. It is possible that the translational stability provided by the facets will break down if traumatically induced vertical instability is not treated. Rotation increased with sectioning (Figure 7A) but remained less sensitive than AVI/PVI.

Threshold selection must ultimately consider clinical intent. In early triage or screening (e.g., post-trauma), maximizing sensitivity may be preferred, while surgical decision-making or impairment determinations may require higher specificity. Although the cadaveric results offer a valuable starting point, larger in vivo or injury-based validation studies are necessary before establishing clinical thresholds.

### 4.4. Radiation and Repeatability Considerations

Flexion-extension radiographs have an extremely low radiation burden (~0.04 mSv), comparable to five days of natural background exposure [62,63]. A low radiation burden is important, particularly in clinical studies that require multiple imaging time points.

Automated measurement reproducibility is essential for harmonized reporting. Although the current study used a single FDA-cleared system, multiple research groups have reported machine-learning/AI methods to obtain vertebral landmarks [64,65,66,67]. The methodology for calculating IVM from vertebral landmarks should be applicable using alternative, sufficiently validated technology. Larger, more demographically diverse datasets will be needed to refine normative ranges and to evaluate population-specific differences.

### 4.5. Limitations

The study has several limitations, including:

- Early or mild degeneration in asymptomatic volunteers could not be fully excluded without an MRI. The asymptomatic cohort may not reflect the full diversity of adult populations.
- Asymptomatic volunteers may flex and extend more than patients. Good patient effort may be essential to detecting true instabilities. Instabilities might be missed due to poor patient effort. This might be mitigated with technician/technologist training and asking patients to first watch a training video (e.g., https://www.youtube.com/watch?v=AWooInuVo1Y accessed on 19 November 2025).
- Normative reference values were derived exclusively from radiographically non-degenerated levels. The generalizability of the metric thresholds to degenerated spines, where the biomechanical context differs, needs further exploration. For instance, a 20% translation may be asymptomatic and acceptable at one stage of degeneration, yet the same magnitude of motion could precipitate symptoms in a severely degenerated or stenotic spine, particularly in the presence of inflammation. The complex interplay between degenerative morphology, motion magnitude, and symptom generation remains poorly characterized.
- Symptom data were unavailable for the pooled preoperative or whiplash-associated exams, limiting clinical correlation. Additional research is required to support the utility of these metrics in guiding treatment decisions.
- The cadaveric model, while valuable for ground-truth injury simulation, cannot replicate the biological complexity caused by real-life trauma or degeneration. The ages of the cadavers were likely older than those of most of the patients studied. The implication of this is not fully understood.
- The 5° minimum rotation threshold was chosen rationally but has not yet been empirically validated.
- Only sagittal plane intervertebral motion was assessed, and the diagnosis of axial and coronal plane motion abnormalities may be important in addition to abnormal coupling between planes.
- Although developed in the context of instability, the IVM metrics may also prove useful for characterizing hypomobility, which warrants dedicated study [68].

Despite these limitations, the combined datasets offer important insights into how instability might be conceptualized, measured, and reported. Before widespread and routine clinical adoption, candidate metrics should be validated as cost-effective in diagnosis and treatment algorithms and applicable across a range of health-care settings. Correlations between symptoms and MRI/CT findings would be part of the validation.

## 5. Conclusions

Using a single FDA-cleared automated analysis pipeline, this study established level-specific normative reference data for cervical intervertebral motion (C2–C7) and then applied those references to estimate abnormality prevalence in two symptomatic clinical cohorts and to test candidate instability metrics in a controlled cadaveric ligament-sectioning model. Normative intervertebral rotation and translation differed significantly by level, and the derived regression relationships between rotation and translation and between rotation and disc height change provided the foundation for standardized, rotation-adjusted instability indices.

Across real-world clinical cohorts, objective IVM abnormalities were uncommon when defined relative to normative limits, and prevalence estimates were strongly influenced by the degree of achieved flexion-extension motion. In pooled preoperative studies, total C2–C7 rotation was substantially lower than in asymptomatic volunteers, which plausibly contributed to the lower prevalence of abnormalities observed. Restricting analysis to examinations demonstrating adequate global motion (>60° C2–C7 rotation) increased the prevalence of several abnormalities, underscoring that inadequate patient effort might mask clinically relevant motion findings and should be treated as a key determinant of test interpretability.

In the cadaveric injury model, sequential sectioning increased intervertebral rotation but produced minimal change in sagittal-plane translation, and the translation-based TI-Index did not change appreciably. In contrast, the anterior and posterior vertical instability indices (AVI/PVI)—which quantify disc height change in the context of rotation—changed dramatically with sectioning and were uniquely sensitive to even the earliest destabilization step. Diagnostic performance analyses demonstrated excellent discrimination for AVI and PVI (AUC 0.96–0.97), with optimal Youden thresholds near 3.1–3.2 yielding high sensitivity (0.89) and perfect specificity (1.00) in a cadaver ligament-sectioning model, whereas translation metrics showed poor discrimination (AUC 0.58).

Collectively, these results support three conclusions applicable to further research: (1) level-specific normative reference data may prove effective in cervical instability assessment, and patient effort should be considered when interpreting IVM data; (2) sagittal-plane translation alone may be an insensitive marker of acute ligamentous compromise under physiologic loading; and (3) regression-based vertical instability indices (AVI/PVI) may be strong candidates for standardized, physiology-guided cervical instability metrics because they demonstrated both mechanistic sensitivity in controlled injury testing and interpretable prevalence patterns in large clinical datasets.

## Figures and Tables

**Table 1:** Means ± standard deviations, and the upper limit of the 95% confidence interval (in parentheses) for radiographically normal levels in 341 asymptomatic volunteers. The rotation difference is the maximum difference in the amount of intervertebral rotation between a level and the adjacent levels. The segment angle difference is the maximum difference between a level and the immediately adjacent levels, measured from the flexion radiographs.

Level	Rotation (deg)	Translation (%EPW)	Translation (mm)	Rotation Difference (deg)	Segment Angle Difference (deg)	N	Rotation (%C2C7)	N (C2–C7)
C2–C3	8.7 ± 2.8 (14.2)	12.1 ± 4.7 (21.2)	1.8 ± 0.7 (3.1)	5.5 ± 2.8 (11.1)	4 ± 3.1 (10)	330	12.1 ± 4.3(20.4)	168
C3–C4	14.1 ± 3.4 (20.9)	15.4 ± 4.7 (24.6)	2.3 ± 0.7 (3.7)	6.5 ± 2.6 (11.6)	5.6 ± 3 (11.5)	301	19.1 ± 4 (26.9)	153
C4–C5	17.9 ± 3.2 (24.1)	18.1 ± 4.5 (26.9)	2.7 ± 0.7 (4.1)	4.8 ± 2.6 (9.9)	4.8 ± 2.8 (10.3)	245	23.9 ± 3 (29.9)	130
C5–C6	19.1 ± 4.5 (27.9)	15.2 ± 4.5 (24.1)	2.4 ± 0.7 (3.8)	3.8 ± 2.6 (8.9)	4.1 ± 2.7 (9.4)	186	24.8 ± 3.5(31.7)	104
C6–C7	16.5 ± 4.7 (25.7)	9 ± 3.1 (15)	1.5 ± 0.5 (2.5)	3.4 ± 2.6 (8.6)	3.9 ± 2.9 (9.6)	122	21.1 ± 4.8(30.6)	122
Avg	14.4 ± 5.3 (24.8)	14.3 ± 5.3 (24.6)	2.2 ± 0.8 (3.7)	5.1 ± 2.9 (10.7)	4.6 ± 3 (10.5)			

EPW = Endplate width.

**Table 2:** R-squared values from linear regressions between intervertebral rotation and intervertebral translation, and between intervertebral rotation and the change in anterior and posterior disc heights.

Level	N	Rotation vs. Translation	Rotation vs. Change in Anterior Disc Height	Rotation vs. Change in Posterior Disc Height
C2–C3	330	0.56	0.85	0.82
C3–C4	301	0.48	0.86	0.83
C4–C5	245	0.3	0.81	0.83
C5–C6	186	0.48	0.91	0.87
C6–C7	122	0.56	0.95	0.91

**Table 3:** Prevalence of abnormalities in the cervical spines of asymptomatic volunteers. These data include all analyzed levels (where rotation >5°), not just the radiographically normal levels used to establish normative reference data. TI = Translational Instability Index; AVI = Anterior Vertical Instability Index; PVI = Posterior Vertical Instability Index; SDH = Standardized disc height [38]; Rot Diff = Rotation Difference between levels; Angle Diff = Segmental angle difference between levels; Trans = Translation.

Level	N	TI > 2	AVI > 2	PVI > 2	SDH < −2	Rot Diff > 11°	Angle Diff > 11°	Trans > 20% EPW
C2–C3	306	4.9	1	2.3	2.3	2.6	2.3	7.2
C3–C4	335	2.1	3	4.2	6.3	6.3	5.4	14.6
C4–C5	333	1.8	5.7	6.6	8.4	3.6	4.5	27.9
C5–C6	300	3	1	5.7	17	3.3	3.3	8.3
C6–C7	167	3.6	0	0.6	12.6	1.8	3.6	0

**Table 4:** Prevalence of abnormalities found in the pooled preoperative patient flexion-extension exams. The acronyms and column headings are the same as in Table 3.

Level	N	TI > 2	AVI > 2	PVI > 2	SDH < −2	Rot Diff > 11°	Angle Diff > 11°	Trans > 20% EPW
C2–C3	1631	1.8	1.2	3	1	0.5	3.1	2.8
C3–C4	1954	0.7	1.3	4	9.5	1.6	6.7	4.3
C4–C5	1924	0.6	2.6	4.3	14.1	4.8	8	6
C5–C6	1518	0.5	0.7	3.6	46.5	5.3	6.5	0.5
C6–C7	1022	0.9	0.7	0.5	28.1	2.6	3.7	0

**Table 5:** Prevalence of abnormalities found in the pooled preoperative patient flexion-extension exams, including only those exams where the C2–C7 rotation was >60 deg. The acronyms and column headings are the same as in Table 3.

Level	N	TI > 2	AVI > 2	PVI > 2	SDH < −2	Rot Diff > 11°	Angle Diff > 11°	Trans > 20% EPW
C2–C3	290	3.1	1.7	5.2	0.7	1	3.4	9.3
C3–C4	300	1.7	2.3	6	4.7	2.7	6	18
C4–C5	300	0.3	3.7	6.7	8	5.7	4.7	24.3
C5–C6	298	0.3	0.3	5.4	35.9	11.7	6	1.7
C6–C7	280	0.7	1.1	1.1	21.8	5.7	2.5	0

**Table 6:** Prevalence of abnormalities among patients with a history of symptoms believed to be related to a motor vehicle accident. The acronyms and column headings are the same as in Table 3.

Level	N	TI > 2	AVI > 2	PVI > 2	SDH < −2	Rot Diff > 11°	Angle Diff > 11°	Trans > 20% EPW
C2–C3	713	4.3	0.4	4.4	2.1	2.7	2.4	6.4
C3–C4	840	1.2	0.8	6.9	12.2	4.5	6.4	9.6
C4–C5	837	1	3.7	6.2	13.5	4.9	5.8	12
C5–C6	686	1	0.7	4.9	24.7	5.7	5.4	3.6
C6–C7	399	3.9	0.8	1.3	17.2	4.6	3.6	0

**Table 7:** Area under the curve, optimal thresholds, and sensitivity and specificity at the optimal threshold for several potential instability metrics.

Metric	AUC	Optimal Threshold	Sensitivity	Specificity
AVI-Index	0.96	3.2	0.89	1
PVI-Index	0.97	3.1	0.89	1
Rotation	0.88	19.8	0.68	1
Translation (%EPW)	0.58	20.5	0.46	0.88
Translation (mm)	0.58	3.1	0.46	0.88
Angle Difference	0.66	4	0.84	0.5
Rotation Difference	0.83	5.8	0.65	0.88

## Data Availability

The imaging datasets presented in this article are not available due to privacy restrictions.

## Abbreviations

The following abbreviations are used in this manuscript:

IVM	Intervertebral motion
QMA	Quantitative Motion Analysis
SDH	Standardized Disc Height
TI-Index	Translational Instability Index
AVI-Index	Anterior Vertical Instability Index
PVI-Index	Posterior Vertical Instability Index
AUC	Area Under the Curve
FECA	Federal Employees’ Compensation Act
AOMSI	Alteration Of Motion Segment Integrity
AMA	American Medical Association
